# Refractive lens exchange in modern practice: when and when not to do it?

**DOI:** 10.1186/s40662-014-0010-2

**Published:** 2014-12-10

**Authors:** Jorge L Alió, Andrzej Grzybowski, Dorota Romaniuk

**Affiliations:** Vissum Corporation, Alicante, Spain; Division of Ophthalmology, Universidad Miguel Hernández, Alicante, Spain; Department of Ophthalmology, Poznan City Hospital, Poznań, Poland; Chair of Ophthalmology, University of Warmia and Mazury, Olsztyn, Poland; Clinical Department of Ophthalmology, Silesian University of Medicine, Katowice, Poland; Avda de Denia s/n, Edificio Vissum, 03016 Alicante, Spain

**Keywords:** Refractive lens exchange, Clear lens exchange, Refractive surgery, Lens surgery, Ophthalmology, Eye surgery

## Abstract

Cataract surgery due to advances in small incision surgery evolved from a procedure concerned with the primary focus on the safe removal of cataractous lens to a procedure focused on the best possible postoperative refractive result. As the outcomes of cataract surgery became better, the use of lens surgery as a refractive modality in patients without cataracts has increased in interest and in popularity. Removal of the crystalline lens for refractive purposes or refractive lens exchange (RLE) presents several advantages over corneal refractive surgery. Patients with high degrees of myopia, hyperopia and astigmatism are still not good candidates for laser surgery. Moreover, presbyopia can currently only be corrected with monovision or reading spectacles.

RLE supplemented with multifocal or accommodating intraocular lenses (IOLs) in combination with corneal astigmatic procedures might address all refractive errors including presbyopia, and eliminate the future need for cataract surgery.

## Introduction

### Historical background of the clear lens extraction

The concept of clear lens extraction dates back to the XVIIIth century, when Abbé Desmonceaux in 1776 was the first to perform such a surgery in France. First systematically conducted operations of clear lens exchange in high myopia in children and young adults were made by Polish ophthalmologist Vincenz Fukala in the last decades of the 19th century in Vienna. Fukala’s operation in myopic patients was widespread among many ophthalmologists in Europe, but due to high rates of post-surgical retinal detachment, the procedure was gradually abandoned at the beginning of the 20th century [1-3].

An intensive development of new concepts and techniques in lens surgery in the 20th century led to clear lens extraction again. The introduction of first posterior–chamber intraocular lens (PC-IOL) by Harold Ridley in 1949 was the first big step in contemporary cataract surgery. In 1952, Baron implanted the first anterior chamber IOL fixated in iridocorneal angle. Then, the concept of lens emulsification with ultrasounds as also the irrigation/aspiration (I/A) technique in phacoemulsification of cataract surgery were developed. Complete and much easier lens removal significantly decreased the number of post-surgical complications. Another huge step in lens surgery was the invention of a foldable intraocular lens (IOL) in the 1980s. This was the beginning of micro-incision cataract surgery (MICS).

## Review

### Surgical techniques for refractive lens exchange

#### General considerations

The surgical technique of refractive lens exchange (RLE) is a variation of standard cataract surgery. The main elements that make a difference between standard cataract surgery and RLE are: clarity of crystalline lens in the absence of cataract, and a presence of abnormal ocular anatomy resulting in a high refractive error, which becomes an indication for RLE. Correction of presbyopia and gaining spectacle-independence in elderly patients with no anatomical ocular pathologies and normal axial length is also an important indication for RLE.

The ideal technical elements of a successful RLE surgery include the following:

- Anatomically minimally invasive surgery with minimal trauma to the corneal endothelium, iris, and other ocular tissues.
- A secure, watertight 2.2 mm (or less) micro incision in clear cornea, optimally 1.0 mm from the limbus, situated at the steepest corneal meridian in order to minimize surgically induced astigmatism or to reduce pre-existing corneal astigmatism.
- Fixation of an appropriate PC-IOL in the capsular bag with low induction of posterior capsular opacification (PCO).

Some special considerations in eyes typically encountered in cases of RLE exist. They are listed below:

- In eyes with high axial myopia, depth and stability of the anterior chamber are abnormal, which necessitates the use of dispersive (heavy) viscoelastic material.
- In eyes with excessive axial length, the risk of perforation during retrobulbar injections [4] is high.
- In short, hyperopic eyes, an increased risk of choroidal effusion and macular edema should be considered.

MICS is an approach to RLE surgery through an incision less than 1.8 mm with the purpose of reducing surgical invasiveness and improving surgical outcomes. MICS favors the use of fluidics, reducing the use of phacoemulsification power. Bimanuality provides an opportunity for easy and comfortable manipulation in the anterior chamber area versus standard coaxial technique. Long term stability of MICS outcomes and having a wide range of surgical capacities make MICS the most modern and adequate approach to minimally invasive RLE surgery.

### Intraocular lens and IOL power selection in refractive lens exchange

The intraocular power calculations for clear lensectomy are not different from standard calculations used for cataract extraction. The patients are usually much younger, however, and the loss of accommodation should be discussed thoroughly [5]. The actual desired postoperative refraction should also be discussed since a small degree of myopia (–0.50 D) may be desirable in the case of monofocal IOL use. Apart from appropriate patient selection, the most important assessment for successful multifocal lens use requires precise preoperative measurements of axial length and accurate lens power calculations. Optical and immersion ultrasound biometry techniques in combination with the Holladay 2 formula can yield accurate and consistent results. When determining lens power calculations, the Holladay 2 formula takes into account disparities in anterior segment and axial lengths by adding the white-to-white corneal diameter and lens thickness into the formula [6].

Addition of these variables helps to predict the exact position of the IOL in the eye and has improved refractive predictability. The SRK-T and the SRK II formulas can also be used as a final check in the lens power assessment, and as for eyes with axial length less than 22.0 mm, Hoffer Q formula should be utilized for comparative purposes (Table 1) [7].

**Table 1:** **Criteria for IOL calculation formula selection depending on axial length of the eye** [6-8]

**Criteria**	**Axial length** **<22.0 mm**	**Axial length 22.0 mm**	**Axial length 24.5 mm**	**Axial length >** **26.0 mm**
**1** ^**st**^ **choice formula**	HOFFER-Q, HAIGIS	SRK-T, HAIGIS	SRK-T, HAIGIS	SRK-T, HAIGIS
**2** ^**nd**^ **choice formula**	HOLLADAY II	HOLLADAY	HOLLADAY	---

The study by Wang and Chang [8] compared different methods of calculating IOL power in eyes with different axial lengths. They found that employing the Haigis formula resulted in the smallest post-operative median absolute error (MedAE) and mean absolute error (MAE). The Haigis formula requires three variables: K - corneal power (measured in radii of curvatures instead of dioptric values), AL – axial length of the eye, and ACD - (phakic) anterior chamber depth.

In a normal cornea, standard keratometry and computed corneal topography are accurate in measuring four sample points to determine the steepest and flattest meridians of the cornea, thus yielding accurate values for the central corneal power. In an irregular cornea, such as one that had undergone prior keratorefractive surgery, these four simple points are insufficient in providing an accurate estimate of the center corneal refractive power [9].

Many different lens equations and strategies exist for post-refractive eyes. Some equations use historical data (pre-operative refraction readings and keratometry) while others do not. Some strategies involve using specialized equipment, like intraoperative aberrometry, Scheimpflug tomography or anterior segment optical coherence topography (OCT), to measure additional corneal parameters that could be helpful in achieving a more accurate lens power calculation.

The inaccurate estimation of corneal refractive power can be attributed to two factors:

A. Inaccurate measurement of anterior corneal curvature by standard keratometry or computerized videokeratography (CVK): Standard keratometry or simulated keratometry from CVK measures only four paracentral points or small regions. This is insufficient for the post-surgical cornea, which can have wide ranges of curvature even within the central 3.0 mm region.
B. Inaccurate calculation of corneal refractive power from the anterior corneal curvature by using the standardized value for refractive index of the cornea (1.3375 in most keratometers and CVK devices): based on the assumption that there is a stable anterior corneal curvature/posterior corneal curvature ratio, the standardized index of refraction was used to convert the measurements of anterior radius of curvature for the estimation of total corneal refractive power. However, procedures that remove corneal tissue [e.g., photorefractive keratectomy (PRK) or laser in situ keratomileusis (LASIK)] change the relationship between the front and back surfaces of the cornea, thus invalidating the use of the standardized refraction index [10].

The choice of using the IOL power calculation method is an important issue in RLE. In long, myopic eyes especially, the problems related to standard IOL power calculation methods are well known.

### Refractive lens exchange in myopia

Patients with high myopia are often willing to have lens refractive procedure performed in order to be made independent from the use of spectacles or contact lenses. Many original studies [11-18] and reviews [19-27] have been made to assess the results of such procedures and risk of intraoperative and postoperative complications. Their results are shown in Table 2.

**Table 2:** **Refractive lens extraction in myopia**

**Author,** **year**	**No. of eyes/** **patients**	**Mean preoperative SE** **(D)** **/axial length** **(mm)**	**Preoperative VA**	**Postoperative VA**	**Postoperative refractive error**	**Follow-** **up-** **time**	**Retinal detachment rate**
Arne, 2004 CLE versus pIOL [11]	36/18 RLE 41/21 pIOL	SE: -16.7 RLE -13.6 pIOL AL: 28.43	N/R	At 1 year: pIOL -78% better than preop RLE - 83.3% better than preop	-1.88 ± 0.83 D RLE -1.06 ± 0.78 D pIOL	47.65 months	2/77 eyes (2.59%) only in CLE group
Emarah, El-Helw, 2010 [12]	28/16 RLE 27/15 ICL	SE: -17.54 RLE -16.45 ICL AL: N/R	CDVA 0.39- RLE group CDVA 0.51- ICL group	RLE group: CDVA 0.61, 71.4% postop UCVA better thanpreop CDVA ICL group: CDVA 0.79, 51.8% postop UDVA better than preop CDVA	SE: -0.99 ± 0.88 D-RLE -0.63 ± 0.86 D-ICL RLE: 82% in goal refraction ICL: 77% in goal refraction both groups 100% within ±2.00 D of goal refraction	17.1 months	none
Gabric, Dekaris et al., 2002 [14]	72/34	N/R	CDVA 0.5 in 72% of eyes	4 years after surgery: UDVA ≥0.5 in 58.3% BCVA ≥0.5 in 83.3%	70.8% - emmetropia 86.5% within ± 1.0 D of goal refraction 95.8% within ± 2.0 D of goal refraction	48 months	1 eye (0.72%)
Horgan et al., 2005 [15]	62/37 RLE	-13.7/29.3	CDVA ≥0.5 in 52% eyes	Mean UCVA 0.3	-1.09 ± 1.34 D	>10 years	2/62 eyes (3.2%)
Ravalico et al., 2003 [18]	388/273, RLE/cataract extraction	-15.95/29.79	CDVA 0.20	CDVA 0.50	SE -2.00 ± 1.62 D	47.16 months	1 eye (0.26%)
Fernandez- Vega et al., 2003 [13]	190/107, RLE	-17.84/26.0	CDVA 0.37	83.7% - better than preop 12.6% - equal to preop 3.7% - worse than preop	Mean -1.22 D 41% -within ± 1.00 D of emmetropia 79% - within ± 2.00 D of emmetropia	4.78 years	2.10%
Guell et al., 2003 [28]	44/30, RLE or initial lens opacification	-15.77/N/R	CDVA ≥0.5 in 63.2% of patients	CDVA ≥0.5 in 82.9%	Mean -1.05 D 52.7% within ± 1.00 D of emmetropia 94.1% within ± 2.00 D of emmetropia	31.45 months	none

SE: spherical equivalent, RLE: refractive lens extraction, VA: visual acuity, UDVA: uncorrected distance visual acuity, CDVA: corrected distance visual acuity, BCVA: best corrected visual acuity, ICL: implantable collamer lens, pIOL: phakic intraocular lens, N/R: not reported.

In a retrospective study [11], phakic IOL implantation and RLE were compared in a group of highly myopic 30-50 year-old patients. RLE was performed in eyes with the anterior chamber shallower than 2.8 mm or at the beginning of presbyopia, whereas phakic IOL was implanted in eyes with no recent visual acuity decrease or presbyopic refraction changes. At one-year follow-up, the results were similar in both groups. In conclusion, phakic lens implantation in myopic patients of 30-50 year-olds was a more adequate refractive technique with lower risk of corrected distance visual acuity (CDVA) loss, and extraction of crystalline lens can be performed as a secondary procedure.

Another study [12] compared RLE and collamer lens (Visian) implantation in patients less than 45 years old with myopia greater than -12.0 D. The RLE group showed better results for postoperative CDVA, and had no serious complications such as retinal detachment (RD), endophthalmitis of inflammatory reaction. In the implantable collamer lens (ICL) group however, lens opacity, pigment dispersion, macular hemorrhage or pupillary block glaucoma occurred. Four eyes from the RLE group required yttrium aluminum garnet (YAG) capsulotomy for PCO.

The issue of different techniques used in RLE in high myopia was also studied. It was shown that considering effective phacoaspiration time, complication rates and intra- and post-operative complications of supracapsular phacoaspiration were safer and presented lower risk of complications than endocapsular phacoaspiration using the divide and conquer technique [29]. The preoperative and postoperative CDVA did not differ significantly between the 2 groups.

In spite of encouraging results of refractive lens surgery in both myopic and hyperopic eyes, there still remains a number of complications that are difficult to avoid [18,30-34].

The most vision-threatening complication of RLE is RD, with incidence from 8.1% [33] to 1.5-2.2% [13,34] (Table 3). In a normal population, RD occurs in 1/8500 eyes [42,43]. The odds of RD however, can increase to 1/850 eyes in cases of myopia greater than -10.0 D in unoperated eyes – 0.68% [44], and in eyes after cataract extraction with IOL implantation. Retinal complications, especially in highly myopic eyes after refractive surgery such as RLE, are mainly attributed to two possible causes: [44] i) higher incidence of predisposing retinal lesions in myopic eyes and ii) a hypothesis that refractive surgery may induce some iatrogenic factors, which can increase the incidence of such pathology. To avoid RD, careful preoperative funduscopic examination with scleral depression should be made to assess the state of the vitreous body. Intraoperatively, the minimal disturbance of intraocular environment is of great importance. Some authors [7] recommend a bimanual microincision phacoemulsification (BMMI) or small–incision lens extraction in myopic eyes [25]. Prophylactic laserotherapy of lattice degeneration in myopic eyes is of modest efficacy and should be avoided instead [13]. During lens surgery, a transient decrease of intraocular pressure (decompression effect) is induced and can cause changes in the vitreous body, especially if vitreous degenerations already exist [44]. Changes detected in proteins of pseudophakic eyes coexist with alterations in structure of the vitreous body. They can contribute to the occurrence of retinal complications after lens surgery.

**Table 3:** **Retinal detachment after RLE in highly myopic eyes**

**Author,** **year**	**Retinal detachment rate (%)**	**Type of study,** **No. of eyes**	**Type of surgery**	**Follow-** **up**
Fernandez-Vega et al. (2003) [13]	2.1	Retrospective, 190	RLE, high myopia	4.78 years
Pucci et al. (2001) [27]	4.0	Retrospective, 25	RLE, high myopia	49.2 months
Neuhann et al. (2007) [32]	1.5-2.2	Retrospective, 2000	RLE, high myopia	>2 years
Lyle and Jin, (1994) [35]	0	Retrospective, 31	RLE, high myopia	20 months
Barraquer et al. (1994) [36]	7.3	Retrospective, 165	RLE, high myopia	31 months
Gris et al. (1996) [37]	2.17	Retrospective, 46	RLE, high myopia	6-15 months
Lee et al. (1996) [38]	0	Retrospective, 31	RLE, high myopia	13 months
Jimenez – Alfaro et al. (1998) [39]	0	Retrospective, 26	RLE, high myopia	12-26 months
Colin et al. (1999) [40]	8.1	Retrospective, 49	RLE, high myopia	7 years
Alio et al. (2007) [41]	2.7	Retrospective, 439	RLE, high myopia	8 years

RLE: refractive lens extraction.

It was argued [21] that in eyes with myopia greater than -8.0 D in pre-presbyopic patients who still accommodate, RLE should not be considered due to increased risk of RD (2.8-8.1%) and phakic lens implantation should be performed in such cases. It was shown in a long-term follow-up study of RLE in high myopia [17], that myopic macular degeneration developed post-surgically in 12 eyes, YAG-capsulotomy for PCO was required in 38/62 eyes, and retinal rhegmatogenous RD in 2/62 eyes.

In the analysis of post-operative RD, two factors are considered important: intraoperative capsular tear with vitreous loss and neodymium-doped yttrium aluminum garnet (Nd:YAG) laser capsulotomy performed for PCO.

Preoperative determination of RD risk, especially in myopic eyes with axial length greater than 26.0 mm and spherical equivalent superior to -6.00 D is of great importance [20]. In young myopic patients, clear lens extraction can induce vitreous changes and increase tractions of the retina, which do not occur in old age after cataract extraction. In a study by Alio et al. [20] The patients were divided into 2 groups according to age (group 1 ≤ 50 years and group 2 > 50 years) and axial length (≤28.0 mm and >28.0 mm). Eyes with longer axial lengths demonstrated higher incidences of RD. This complication was also more frequent in younger patients (3.65% in group 1 comparing to 2.52% in group 2, p < 0.05). The risk factors of post-RLE RD include [44]: increased axial length, age less than 50 years, male sex, Caucasian race, peripheral retinal degenerations, intraoperative rupture of the posterior capsule, and Nd:YAG capsulotomy for PCO.

A combined operation of pars plana vitrectomy with clear lens extraction in correction of high myopia in patients can also be considered to reduce the risk of RD [45].

Among frequent post-operative complications, RLE may be followed by cystoid macular edema in the first few weeks after surgery. The greatest risk of this complication is carried when the procedure is done for correction of high myopia. RLE in highly myopic eyes is recommended after detachment of vitreous body, which diminishes the risk of RD.

A long-term complication of RLE procedure is PCO. It can develop months to years after the surgical procedure. YAG capsulotomy can be more risky in myopic eyes. Each millimeter of increased axial length increases the risk of RD after YAG capsulotomy by a factor of 1.5 [46]. No preoperative prophylaxis can be made however, there are a number of intraoperative methods that can be used to reduce the incidence of PCO including the capsulorrhexis overling the edge of the IOL optic, cortical cleaving hydrodissection, meticulous cortical clean-up and the implantation of a sharp posterior edge IOL. As much as possible, YAG capsulotomy should be avoided [47]. Another complication of RLE might be a worsened twilight vision with halo perception and glare after the implantation of multifocal IOL [48].

The less common complications include choroidal neovascularization (CNV) formation [49]. CNV formation after clear lens extraction was reported in all patients with preoperative macular lacquer cracks [13], but the presence of myopic CNV in the fellow eye was also a risk factor for the operated eye [49]. There is no clear explanation as to why eyes undergoing RLE are more susceptible to early age-related macular degeneration (AMD) occurrence with or without CNV formation [44], but inflammatory mediators associated with biochemical environmental changes within the eye such as increased free radicals or growth factors can play important roles.

When not to perform RLE in myopic eyes:

- Eyes with advanced peripheral lattice degenerations
- Young eyes with no posterior vitreous detachment
- Laquer cracs in high myopia or myopic CNV in the fellow eye
- Presbyopic eyes with macular degeneration beginning in the fellow eye

### Refractive lens exchange in hyperopia

Small, hyperopic eyes with shallow anterior chambers are more predisposed to closed-angle glaucoma. This makes even moderate hyperopia an indication for RLE, offering very good benefit/risk ratio [47]. In elderly patients where accommodation is weakened despite the clear crystalline lens, its removal with multifocal IOL implantation can be a good option in the absence of other ocular pathologies.

In several studies [50-55], shown in Table 4, satisfactory refractive results were reported in the treatment of hyperopia with RLE. Occurrences of complications such as RD or cystoid macular edema (CME) are lower than in RLE for the treatment of myopia.

**Table 4:** **Refractive lens exchange in hyperopia** (**visual acuities in decimal values**)

**Author,** **year**	**No. of eyes/** **patients/** **mean age** **(years)**	**Mean follow-** **up**	**Preop SE** **(D)**	**Postop SE** **(D)**	**Preoperative UDVA**	**Preoperative CDVA**	**Postoperative UDVA**	**Postoperative CDVA**
Siganos, Pallikaris, 1998 [50]	35/21/N/R	5 years	+9.19 ± 0.34 (distance) +10.41 ± 0.32 (near)	At 1 year: +0.02 ± 0.80 (distance) +1.87 ± 0.31 (near)	Count fingers – 31 eyes, 0.1 VA obtained in 4 eyes	Mean CDVA 0.94 ± 0.014 (spectacle-corrected), 1.037 ± 0.22 (contact lens-corrected)	0.8 (0.5 to 1.0)	91.4% within ±1.00 D of target refraction
Fink et al., 2000 [51]	50/29/, Group A: (preop SE ≤ +4 D), 61.9 Group B: (preop SE > +4 D) 54.7	10 months	Group A: SE: +2.26 ± 0.94 Group B: SE: +6.32 ± 1.32	Group A: -0.18 ± 0.73 Group B: -0.19 ± 1.28	Group A: 0.19 Group B: 0.05	Group A: 1.13 Group B: 1.04	Group A:0.81 ± 0.30 Group B:0.58 ± 0.33	Group A: 1.10 ± 0.17 Group B: 1.02 ± 0.16
Alfonso, et al., 2009 RLE after previous hyperopic LASIK [52]	41/23/51.03	6 months	Pre-IOL SE: Mean +1.32	Mean postop SE: -0.064 ± 0.513	UDVA: 0.189 ± 0.175 logMAR	CDVA: 0.049 ± 0.071 logMAR Mean efficacy index: 0.87 Mean safety index: 1.00	UDVA: 0.113 ± 0.101 logMAR ≥0.5: 97.56% ≥0.8: 58.54%	CDVA: 0.046 ± 0.063 logMAR All eyes within ±1.25 D of target refraction, 73.17% - within ±0.50 D
Preetha Goel, Patel et al., 2003 [53]	20/12/35.75	16.96 months (6 to 35 months)	+6.66 ± 2.17 (+4.75 to +13.0)	+0.68 (0 to +2.50)	0.10 ± 0.09 (0.03 to 0.25)	0.53 ± 0.29 (0.10 to 1.00)	0.45 ± 0.25 (0.10 to 1.00)	0.63 ± 0.30 (0.10 to 1.00)
Pop, Payette 2004 [54] Artisan phakic lens versus RLE + IOL	19/12/20 - Artisan 19/15/36 - RLE + IOL	5.4 months	Artisan: +2.75 to +9.25 RLE + IOL: +2.75 to +7.50	2 months postop: Artisan: 78% within ±1.00 D of emmetropia RLE + IOL: 91% within ±1.00 D of emmetropia	≥0.8: 0 in both groups ≥0.5: 0 in both groups Preoperative UDVA of both groups is not reported in details.	Artisan: 84% >0.8 RLE + IOL: 68% >0.8	2 months postop: ≥0.8: 67% Artisan 64% RLE + IOL ≥0.5: 89% Artisan 82% RLE + IOL	N/R
Koladhouz – Isfahani et al., 1999 [55]	18/10/N/R	10.5 months	+6.17	-0.21	0.06	0.80	0.50	0.80, 39% within ±1.00 D of target refraction

RLE: refractive lens extraction, SE: spherical equivalent, UDVA: uncorrected distance visual acuity, CDVA: corrected distance visual acuity, IOL: intraocular lens, N/R: not reported.

A study by Preetha et al. [53] evaluated the safety, efficacy and predictability of clear lens extraction with PC-IOL implantation in 20 eyes of 12 hyperopic patients. The efficacy index [mean postoperative uncorrected distance visual acuity (UDVA)/mean preoperative corrected distance visual acuity (CDVA)] was 0.84 and the safety index (mean postoperative CDVA/mean preoperative CDVA) was 1.1. No intraoperative complications occurred, although the main postoperative complication was PCO.

Hoffman et al. [56] reported the successful results of bilateral RLE with the use of multifocal AMO Array lens in 50 hyperopic and presbyopic patients. Ninety-two percent of patients gained 20/40 and Jaeger 4 print, and all patients were able to read 20/40 and Jaeger 5 print.

Another study [57] compared RLE with pseudophakic IOL implant and phakic Artisan iris-claw IOL in the treatment of hyperopia. At 1 month postoperatively, the uncorrected visual acuity (UCVA) of the RLE group was slightly better than the Artisan group in that the results were inversed at 2 months postoperatively. Mean endothelial cell loss at 1 year after surgery was 8-10% after phacoemulsification, whereas in the Artisan group 6 months after phakic implantation it was 2.3%. No RD was observed in both groups.

RLE with spherical diffractive IOL implantation in eyes after hyperopic LASIK also revealed to be safe, effective and predictable [52].

Comparing the magnitude of wavefront aberrations in eyes after hyperopic LASIK and RLE [45], the results presented as follows: in the LASIK group, postoperative refraction change significantly correlated with total, corneal and internal RMS-HOA (root-mean-square higher-order aberrations) and spherical aberrations. For the RLE group, no such correlations were noted. RLE was revealed then to be much safer and a better refractive procedure for minimizing total higher order optical aberrations which occur after hyperopic refractive surgery.

RLE can be used to treat hyperopia in extremely short, nanophthalmic eyes or eyes of patients who underwent laser refractive surgery and require correction of remaining hyperopia. In these cases, an accurate axial length measurement to achieve emmetropia is essential [52,58].

Good refraction results were described [59] in highly hyperopic eyes (+7.5 D both eyes) after RLE with IOL implantation in patients with congenital systemic syndromes associated with severe developmental delay who do not cooperate and cannot wear spectacles.

Apart from well-known cataract surgery complications, the risk of complications in short, hyperopic eyes (axial length <21.0 mm) is mostly due to anatomical conditions – less space in the anterior segment and shallow anterior chamber predispose to pupillary block or secondary postoperative intraocular pressure (IOP) increase. The postoperative uveal effusion is also seen more often in hyperopic eyes [30].

RLE in hyperopic eyes (unilateral or bilateral) can be considered in:

- Beginning presbyopia with weakened accommodation of crystalline lens
- High order aberrations, when laser surgery need to be avoided
- High hyperopia in patients with congenital systemic condition who are unable to wear spectacles or contact lenses

### Refractive lens exchange in children

So far only a few studies have been made on this subject, and refractive surgery with the use of excimer laser was more commonly investigated. However, excimer laser correction of refractive errors is limited to those between +6.0 and -12.0 D [60]. Refractive errors beyond this range require other methods of correction.

In children, main indications for refractive surgery are severe anisometropia and severe bilateral ametropia [61].

According to Tychsen et al. [60] one of the major recommendations for pediatric RLE in non-compliant children is a shallow anterior chamber (<3.2 mm where phakic IOL is impossible or too risky to be implanted). The most frequent complication of such surgery is late aphakic/pseudophakic RD, estimated for about 3%. Barrier diode laser may be applied in eyes whose axial length exceeds 29.0 mm, in order to avoid or reduce RD.

A special group of children candidates for RLE are those with high myopia and neurobehavioral disorders, who are non-compliant for wearing of spectacles or contact lenses. It was however postulated that RLE in highly myopic eyes doubles the risk of this complication and has about 30% risk of developing glaucoma [15]. The results of pediatric RLE studies are shown in Table 5.

**Table 5:** **Refractive lens extraction in children**

**Author,** **year**	**No. of eyes/** **patients**	**Mean preoperative SE** **(D)**	**Mean preoperative VA**	**Mean postoperative VA**	**Goal refraction** **(D)**	**Myopic regression** **(D/** **year)**	**Mean follow**-**up time**
Tychsen et al., 2006 [58]	26/13 bilateral	-19.1	0.26 BCVA	0.52 CDVA	0 to +3.0	0.16	4.5 years
Ali et al., 2007 [62]	7/7 unilateral	-16.7	20/2550, UDVA	0.15, UDVA	0 to +4.0	0.43	3.8 years
Bhattacharjee et al., 2010 [63]	2/1 bilateral microsphrophakia, angle closure glaucoma	-23.0 sph -1.0 cyl ax 80 (RE) -24.0 sph -1.0 cyl ax 100 (LE)	0.33 RE, CDVA, 0.25 LE, CDVA	1.0 both eyes	emmetropia	None, After 1 year UDVA 1.0 in both eyes	1 year

SE: spherical equivalent, VA: visual acuity, UDVA: uncorrected distance visual acuity, CDVA: corrected distance visual acuity, BCVA: best corrected visual acuity, RE: right eye, LE: left eye.

Depending on preoperative refractive error, some congenital lens abnormalities (microspherophakia, high myopia/hyperopia, secondary glaucoma) with corresponding refractive error can be an indication for RLE with or without IOL implantation [63].

RLE can serve also in less common situations for e.g. as a method of correcting persistent accommodating spasms after head trauma [64].

Modern indications for pediatric RLE include:

- High anisometropia or severe bilateral ametropia
- Congenital conditions disabling proper binocular vision
- Non-compliant children with high refractive errors where treatment with refractive laser surgery is impossible

### Refractive lens exchange in astigmatism

Previously, in cases of residual astigmatism after cataract surgery or RLE, the only solution was additional corneal laser surgery – LASIK, PRK or soft contact lenses if the correction was still inadequate [65]. Pop et al. [54] studied PRK and LASIK after RLE with spherical IOL implantation for hyperopia or astigmatism. A laser procedure was performed to correct residual ametropia after the first surgery. Both methods of laser correction showed to be equally effective. RLE with IOL implantation was more risky in terms of sight-threatening complications than refractive surgery alone. In selected cases of hyperopic eyes after RLE with residual ametropia however, laser adjustment can be appropriate.

The invention of toric IOLs by Shimizu in 1992 became a milestone in the treatment of astigmatism. Posterior chamber toric lens implantation is a new, highly predictable surgical option for patients with pre-existing corneal astigmatism. A study by Sun et al. [66] evaluated the results of toric IOL implantation for cataract extraction and RLE in 130 eyes of patients with pre-existing astigmatism. In the control group of 51 eyes with pre-existing astigmatism of similar degrees, an implantation of spherical IOL combined with limbal relaxing incisions was performed. In both groups, a significant decrease of refractive astigmatism was achieved. There was no significant superiority of one method over another. The number of eyes with residual astigmatism of 0.75 D or less was larger in the toric IOL group.

Another prospective study [67] performed a RLE in astigmatic eyes with implantation of an AcrySof Toric IOL to correct the preoperative regular corneal astigmatism, varying from 1.0 to 4.0 D. After surgery, the mean reduction of astigmatism was 80%. Good rotational stability was achieved – no eye needed a repositioning of IOL. No eye had any kind of complications, neither intraoperative nor postoperative.

The results of studies concerning RLE in the correction of astigmatism are shown in Table 6.

**Table 6:** **Refractive lens extraction in astigmatism with implantation of toric IOLs** (**visual acuities is decimal values**/ **decimal scale**)

**Author,** **year**	**No. of eyes/** **patients/** **mean age** **(years)**	**Mean follow-** **up** **(months)**	**Keratometry** **(K1,** **K2)** **(D)/** **Axial length** **(mm)/** **Mean IOL power** **(D)**	**Preop SE/** **mean defocus equivalent/** **mean refractive cylinder** **(D)**	**Postop SE/** **mean defocus equivalent/** **mean refractive cylinder** **(D)**	**Preoperative** **CDVA**	**Postoperative** **UCVA**	**Postoperative,** **CDVA**
Sun et al., 2000 (retrospective) [66]	Toric group: 130/99/72 122 eyes – cataract surgery 8 eyes – RLE Non-toric group: 51/45/ N/R	6.9	N/R K2-K1 = 2.59 ± 0.82 (total)	Mean refractive cylinder preop: Toric group: 2.57 ± 1.15 Non–toric group: 2.58 ± 0.89	Toric group: Mean SE: -0.51 ± 0.65 refractive cylinder: -1.03 ± 0.79 Non-toric group: Mean SE: -0.52 ± 0.78 Refractive cylinder: -1.49 ± 0.75	The authors only mention preop UCVA: Toric group: 0.74 ± 0.25 logMAR Non-toric group: 0.78 ± 0.30 logMAR Preop BCVA – N/R	Toric group: 84% of eyes ≥0.5 Non-toric group: 76% of eyes ≥0.5	The authors only mention postop UCVA: Toric group: 69% ≥0.66 Non-toric group: 70.3% ≥0.66 Postop BCVA- N/R
Jaimes et al., 2011 (retrospective) [68]	19/13/48.15 12- keratoconus 1- pellucid marginal degeneration	7.89	Mean K readings: 46.31 ± 3.39 AL – N/R IOL: AcrySof ToricSN60T3-T9 mean power =14.9	Sphere: -5.25 ± 6.40 Cylinder: +3.95 ± 1.30 SE refraction: -7.10 ± 6.41	Sphere: +0.22 ± 1.01 Cylinder: +1.36 ± 1.17 SE refraction: -0.46 ± 1.12	CDVA: 0.28 ± 0.55 logMAR	Postop UDVA 0.29 ± 0.23 logMAR	CDVA: 0.11 ± 0.12 logMAR
Leccisotti, 2006 [69]	34/20/56.7	17.4	N/R	-11.0 ± 4.65 /12.0 ± 4.64/1.86 ± 1.39	-1.31 ± 1.08 /1.94 ± 1.57/1.22 ± 1.37	0.55 ± 0.23	0.48 ± 0.25	0.76 ± 0.23 at 12 months
Ruiz-Mesa et al., 2009 (prospective) [67]	32/19/60.1	6	K1 = 43.47 ± 1.20 K2 = 45.75 ± 1.41 AL =23.54 ± 2.04 IOL power =20.87 ± 5.99	Mean refractive sphere: -0.70 ± 5.32 Mean refractive cylinder: -2.46 ± 0.99	Mean refractive sphere: -0.007 ± 0.61 Mean refractive cylinder: -0.53 ± 0.30	0.87 ± 0.10	0.89 ± 0.09, 56.3% of eyes ≥1.00 84.3% of eyes ≥0.80	0.95 ± 0.09
Pop et al., 2001 (retrospective) [70]	65/55/N/R RLE for hyperopia 31 eyes – retreated with PRK 34 eyes- retreated with LASIK	12	Mean K readings: 43.23 ± 1.92 Mean AL: 21.33 Mean IOL power: 32.7 ± 6.0	Preop SE: 3.1% of eyes within ±0.5 D of emmetropia 4.6% of eyes within ±1.0 D of emmetropia	96% of eyes within ±2.0 D of emmetropia 79% of eyes within ±1.0 D of emmetropia 51% of eyes within ±0.5 D of emmetropia	18.5% of eyes ≥1.0 before RLE 60.9% of eyes ≥1.0 before laser adjustment	Postop UDVA: 85% of eyes ≥0.5 46% of eyes ≥1.0	CDVA at 12 months: 100% ≥0.5 after PRK 95.7% ≥0.5 after LASIK

RLE: Refractive lens extraction, SE: spherical equivalent, UDVA: uncorrected distance visual acuity, CDVA: corrected distance visual acuity, AL: axial length, IOL: intraocular lens, PRK: photorefractive keratectomy, LASIK: laser in situ keratomileusis, N/R: not reported.

The issue of RLE for correcting a myopic spherical error or RLE with toric lens implantation associated with stable keratoconus in stage I and II was also undertaken [33,46]. Keratoconus laser refractive surgery or phakic IOL implantation has only a limited value and is also associated with a reduction in corneal endothelial cell density.

The important potential complication of RLE for the correction of astigmatism to be avoided is a mistake in IOL power calculation, which results in a postoperative refraction that is different from the target refraction [30]. As patients’ expectations in this type of surgery are higher than those after cataract extraction, the surgeon must be extremely thorough while choosing the calculation formula and the type of IOL to be implanted.

When considering RLE in astigmatic eyes, the surgeon must be conscious of unpredictability of the results and more often than not, only a slight improvement of the patient’s vision. It is not the preferable method of correction of cylindric errors. All the other possibilities must be introduced to the patient preoperatively.

### Refractive lens exchange in presbyopia

In recent years, several presbyopia-correcting IOLs using accommodating or multifocal designs have been developed. The aim is to restore distance, near, and intermediate visual functions after cataract surgery [5,71].

Multifocal IOL optical designs attempt to provide patients with spectacle independence for distance and near visual conditions by generating several foci at different distances [5,72]. The ability of multifocal IOLs to improve the near visual function in pseudophakic patients has been confirmed by several studies [5,73-76]. This provides near vision at the expense of reducing contrast sensitivity and causing photic visual phenomena, such as increased glare and halos [56,77-80]. Special considerations should be made when a surgeon plans to implant a multifocal IOL in patients with high ametropia as magnification and minimization of the retinal image in myopic and hyperopic patients respectively, may play a significant role in the visual outcome [81].

Accommodating IOLs were designed to mimic the physiologic mechanism of accommodation to avoid the optical side effects of multifocal IOLs. Several mechanisms have been described for this kind of IOL [5,82]. One is the axial (backward and forward) movement of the IOL optic where the ciliary muscle contracts and relaxes. The efficiency of the single-optic principle is dependent on the optical power of the displaced IOL, providing limited near vision [72]. To circumvent this, a dual-optic IOL was designed with a high plus power moving optic coupled with a low-power static minus lens; the two are joined by a spring haptic [83] (Crystalens HD IOL, Bausch & Lomb). The biconvex single-optic accommodating IOL is of a biocompatible third-generation silicone (Biosil) with a refractive index of 1.428. It has a central bispheric modification that was designed to increase depth of focus for better intermediate focus and near focus [Synchrony (Visiogen, Inc.)]. The single-piece silicone IOL has 2 main components (anterior and posterior). Each component has the general design of a plate-haptic silicone IOL; a bridge with a spring function connects the 2 components. The anterior IOL component has a high plus power beyond that required to produce emmetropia. The posterior IOL component has a minus power to return the eye to emmetropia. Once the IOL is in the capsular bag, the tension of the bag compresses the optics. This leads to strain energy in the haptics that is released when there is an attempt to accommodate.

In a comparative study between multifocal and accommodating IOLs [84], it was found that both IOLs can successfully restore the distance visual function after cataract surgery and provide an improvement in near vision. Of note, the low add power of the multifocal refractive IOL provides a wide range of focus, especially in intermediate vision, and provides better near-vision outcomes. Both IOL models had limitations in providing complete near-vision outcomes. The low add multifocal IOL could possibly be a better option for patients with significant intermediate-vision demands.

### Modern indications for refractive lens exchange with a balanced risk/benefit ratio

Our current ability to achieve emmetropia following refractive lens surgery rivals the results of corneal refractive surgery, yet it covers a much wider range of refractive errors. While phakic refractive lenses extend the range of correction for younger patients, RLE also offers, with new IOLs, a high probability of achieving functional binocular vision at distance, intermediate and near focal lengths. For these reasons, RLE will become the dominant refractive procedure for patients past the age of presbyopia. With RLE, patients can enjoy a predictable refractive procedure with rapid recovery, which addresses all types of refractive errors, including presbyopia, and as a bonus they will never develop cataracts [6].

Desire for a life free of spectacle and contact lens correction is not limited to low and moderate myopes under the age of 40. The high myope with an accommodative reserve may be a good candidate for phakic refractive lens implantation, and the presbyopic hyperope has become well recognized as a candidate for RLE with an accommodating or multifocal IOL [33]. A myope over the age of 45, however, may be greeted with skepticism. Surgeons worry that presbyopic low myopes may be unsatisfied with a simple trade off – distance correction for near after bilateral LASIK or a compromise of depth perception with monovision – since multifocal or accommodating IOL may not offer the same quality of near vision they already have without correction. RLE for moderate to high myopes may raise concerns about significant complications, especially RD. In particular, eyes with long axial length and vitreoretinal changes consistent with axial myopia may be at higher risk for RD following lens extraction and IOL implantation.

A study that analyzed trends in refractive surgery in Germany over a 3-year period [85], showed that the predominant type of laser refractive surgery was LASIK, but RLE remained one of the most common non-corneal procedures, and in fact is more popular than phakic IOLs. The refractive surgery style in Germany is comparable to trends in other European countries. Moreover, it was argued that RLE provides greater depth of focus than phakic lenses through the use of multifocal and accommodative lenses. A comparative study of phakic IOLs and RLE [23] underlined that selection between these two procedures depends on various factors, such as a patient’s age, expectations, lifestyle and personality. It was suggested that in younger (<55 years of age) and myopic patients, RD following the procedure must be of concern and it is the best to perform RLE in patients with complete posterior vitreous detachment. Moreover, in hyperopic patients, RLE is a procedure of choice mostly because of anatomic dimensions of their eyes.

Since these are entirely elective procedures, minimizing risk is critical to the success of RLE and refractive surgery in general. Several conclusions emerge from the literature on RD following RLE [32,34].

First, careful preoperative examination and counselling should precede any decision to operate. Complete funduscopic examination with scleral depression and determination of the state of the vitreous body comprise essential steps in the examination. Referral to a vitreoretinal specialist should be entertained if any doubt emerges concerning the nature of a lesion or the indication for prophylaxis.

Second, surgical principles should emphasize minimal disturbance of the intraocular environment. Microincisional techniques facilitate the maintenance of a stable chamber, construction of a round and centered capsulorrhexis [continuous curvilinear capsulorrhexis (CCC)], effective cortical cleaving hydrodissection, efficient aspiration of lens material without application of ultrasound energy, and safe bimanual cortical clean-up through two paracentesis-type incisions. A fresh temporal clear corneal incision may be constructed for introduction of the IOL. All incisions should be Seidel negative at the conclusion of the case [6,7,22].

Third, eventual YAG capsulotomy should be avoided if possible. The construction of a capsulorrhexis that completely overlies the edge of the IOL optic, together with the use of cortical cleaving hydrodissection, meticulous cortical clean-up, and the implantation of an IOL with a sharp posterior edge, all facilitate maintenance of a clear posterior capsule. By following these guidelines, we may be able to obtain the highest benefits with the least possible risks [6,7].

## Conclusions

In conclusion, RLE is an elective intraocular surgery that needs to be minimally invasive, and performed with precision and high accuracy. The indication of this surgery is the presence of high refractive error in the absence of cataract and requires an approach with the risk–benefit ratio in mind depending on the age, refractive condition and pre-operative condition. In general, RLE should be performed only in presbyopic eyes. The main challenge involved is to reach emmetropia. With the rapid recovery and astigmatically neutral incisions currently used for modern cataract surgery, this procedure can be done with greater predictability [47]. For restoration of near, intermediate and far vision, multifocal IOLs are currently superior to available accommodating IOLs [84]. Successful integration of RLE into the general ophthalmologist’s practice is fairly straightforward if the surgeon is following the modern methods of minimally invasive, small incision cataract surgery.
